# The Association of dp-ucMGP with Cardiovascular Morbidity and Decreased Renal Function in Diabetic Chronic Kidney Disease

**DOI:** 10.3390/ijms21176035

**Published:** 2020-08-21

**Authors:** Stefanos Roumeliotis, Athanasios Roumeliotis, Aikaterini Stamou, Konstantinos Leivaditis, Konstantia Kantartzi, Stylianos Panagoutsos, Vassilios Liakopoulos

**Affiliations:** 1 Division of Nephrology and Hypertension, 1st Department of Internal Medicine, AHEPA Hospital, School of Medicine, Aristotle University of Thessaloniki, 54636 Thessaloniki, Greece; st_roumeliotis@hotmail.com (S.R.); a_roumeliotis@hotmail.com (A.R.); konleiv@windowslive.com (K.L.); 2 Department of Microbiology, AHEPA Hospital, School of Medicine, Aristotle University of Thessaloniki, 54636 Thessaloniki, Greece; katerina_stms@yahoo.gr; 3 Department of Nephrology, School of Medicine, Democritus University of Thrace, 68100 Alexandroupolis, Greece; kokan0910@gmail.com (K.K.); spanagou@med.duth.gr (S.P.)

**Keywords:** cardiovascular disease, chronic kidney disease, dephosphorylated uncarboxylated matrix Gla protein, diabetic kidney disease

## Abstract

We aimed to investigate the possible association of the inactive, dephosphorylated, uncarboxylated matrix Gla protein (dp-ucMGP) with oxidized low-density lipoprotein (ox-LDL) and all-cause/cardiovascular (CV) mortality and renal function in diabetic chronic kidney disease (CKD). Ox-LDL and dp-ucMGP were determined in 66 diabetic CKD patients. All patients were prospectively followed for seven years, or until the occurrence of death, or a composite renal outcome of 30% estimated glomerular filtration rate (eGFR) reduction or progression to end-stage renal disease (ESRD) requiring dialysis occurred. Secondary outcomes were the occurrence of CV events. Kaplan–Meier curves showed that patients with plasma dp-ucMGP levels above the median (≥656 pM) had a significantly higher risk for all study endpoints. After adjustment for several well-known cofounders, multivariate Cox analysis showed that high plasma dp-ucMGP levels were associated with all-cause mortality (Hazard ratio-HR = 2.63, 95% Confidence Interval-CI = 1.17–5.94, *p* = 0.02), CV mortality (HR = 2.82, 95% CI = 1.07–7.49, *p* = 0.037) and progression of CKD (HR = 4.02, 95% CI = 1.20–13.46, *p* = 0.024). Circulating dp-ucMGP is associated with mortality and decreased renal function in diabetic CKD.

## 1. Introduction

Cardiovascular (CV) disease is highly prevalent and accounts for more than 50% of all deaths in chronic kidney disease (CKD) patients [1]. Patients in the early stages of CKD are more likely to suffer a fatal CV event than undergo hemodialysis (HD) due to end-stage renal disease (ESRD) [2]. On the other hand, a history of CV disease is associated with a 29% greater risk for the deterioration of kidney function in CKD patients [3]. Therefore, CV disease and CKD are two tightly interrelated conditions. This might be attributed to the high prevalence of endothelial dysfunction, arterial calcification, and stiffness, which predispose to CV disease, progression of CKD, and sudden death. It is now clear that vascular calcification (VC) is the result of the imbalance between promoters and inhibitors of arterial calcification, with the former overwhelming the latter [4]. In uremia, inhibitors of VC are downregulated, with low circulating levels and activity, resulting in accelerated VC and atherosclerosis.

One of the strongest inhibitors of VC is matrix Gla protein (MGP), a small, 10 kD protein, synthesized by smooth muscle cells of the arterial wall and chondrocytes [5]. To become active, MGP needs to undergo vitamin K-dependent carboxylation and phosphorylation [6]. Dephosphorylated, uncarboxylated MGP (dp-ucMGP), is the fully inactive form of the protein that reflects vitamin K deficiency [7,8]. Dp-ucMGP plasma levels have been reported to increase, along with deterioration of renal function in CKD populations [7,9]. Although there is a growing body of evidence suggesting an association of circulating dp-ucMGP with multiple markers of endothelial dysfunction and VC (including carotid intima-media thickness, pulse-wave velocity and coronary calcification score) in the CKD setting, the data regarding the direct association of this VC inhibitor with clinical hard end-points (such as mortality and CV morbidity) remains very limited [7,8]. Dp-ucMGP has been repeatedly associated with dyslipidemia, but no study so far has ever investigated the possible interaction of low density lipoprotein (LDL) oxidation [an early marker of oxidative stress (OS)], endothelial dysfunction, and atherosclerosis) with dp-ucMGP. Furthermore, although there are some limited data in the literature suggesting a possible association between dp-ucMGP and surrogate markers of renal function—such as estimated glomerular filtration rate (eGFR) and proteinuria—until now, no study has examined the possible association between dp-ucMGP and a well-described renal outcome in patients with diabetic CKD.

In this study, we aimed to investigate the association between dp-ucMGP and mortality, CV disease, and decreased renal function in a cohort of patients with diabetic CKD. Moreover, we assessed the possible association between dp-ucMGP and oxidized LDL(ox-LDL).

## 2. Results

The anthropometric, biochemical and clinical characteristics of diabetic CKD patients according to median plasma dp-ucMGP are shown in Table 1. Although the two groups did not differ significantly in age, gender, body mass index (BMI), hemoglobin, glycated hemoglobin (HBA1c), duration of type 2 diabetes mellitus (T2DM) and hypertension, patients in the high dp-ucMGP group had significantly lower serum albumin, higher proteinuria levels, and lower eGFR at baseline. Compared to the low group, patients in the high dp-ucMGP group had higher triglycerides, lower high-density lipoprotein (HDL) cholesterol, and higher C-reactive protein (CRP) levels. No differences were found in total, LDL or ox-LDL cholesterol among the two groups. Previous history of CV disease did not differ in the two groups. Table 2 shows the correlation matrix analysis between circulating dp-ucMGP and several characteristics of the patients studied. Dp-ucMGP was negatively correlated with eGFR, both at baseline and after the follow-up period, and with the change in eGFR (*r* = −0.69, *p* < 0.0001, *r* = −0.72, *p* < 0.0001 and *r* = −0.51, *p* < 0.0001, respectively). A significant positive correlation was found between dp-ucMGP, CRP (*r* = 0.28, *p* = 0.03), proteinuria (*r* = 0.40, *p* = 0.002), and triglycerides (*r* = 0.28, *p* = 0.03). No association was found between plasma dp-ucMGP and total /LDL/HDL or ox-LDL cholesterol.

**Figure 1:**
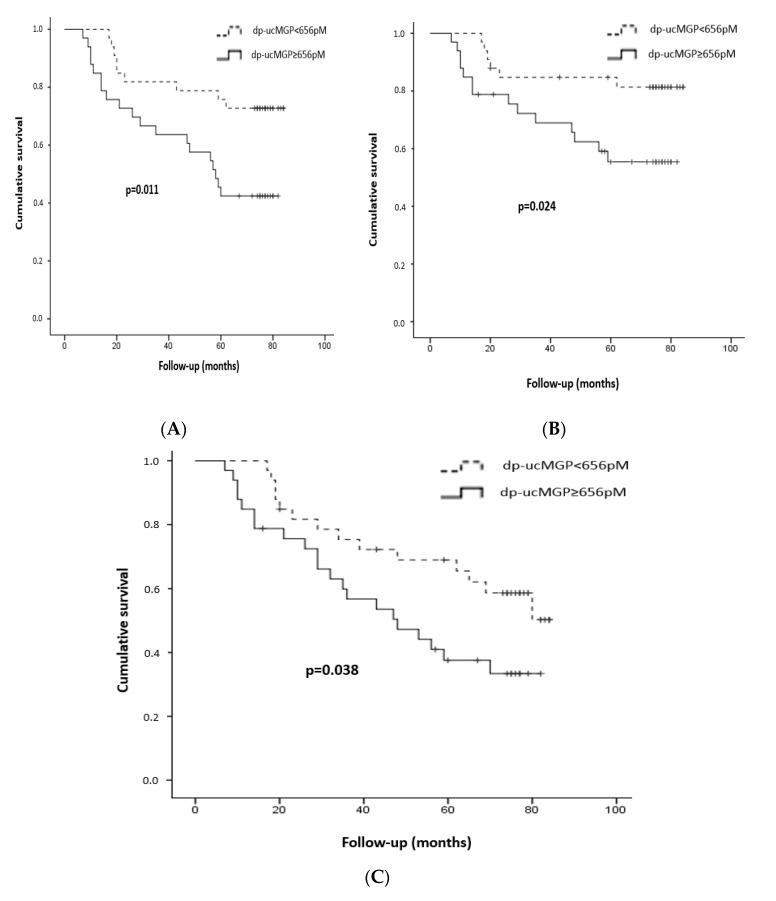
Kaplan–Meier curves for all-cause mortality (**A**), cardiovascular mortality (**B**) and cardiovascular events (**C**) in patients with high and low plasma levels of dp-ucMGP [according to the median value (656 pM)]. Log-rank test *p* = 0.011, 0.024 and 0.038, respectively.

After seven years of follow-up, 28/66 patients died (19 were in the high dp-ucMGP group); 20/66 died from CV disease (14 in the high group) and 35/66 experienced a new fatal or non-fatal CV event (21 in the high group). Moreover, 24 patients presented the composite outcome of deterioration of renal function (four in the low and 20 in the high dp-ucMGP group). Kaplan–Meier curves (Figure 1) showed that patients with plasma dp-ucMGP levels above the median (≥656 pM) presented a significantly higher risk for all-cause/CV mortality and CV events compared to the low dp-ucMGP group (*p* = 0.011, *p* = 0.024, *p* = 0.038, respectively, log-rank test).

Univariate Cox proportional hazard analysis revealed that high circulating dp-ucMGP levels were independently associated with all-cause [Hazard ratio (HR) = 2.68, 95% Confidence Interval (CI) = 1.21–5.94, *p* = 0.015)]/CV mortality (HR = 2.86, 95% CI = 1.10–7.47, *p* = 0.031), CV events (HR = 2.03, 95% CI = 1.02–4.02, *p* = 0.043) and ≥30% eGFR reduction or progression to ESRD (HR = 7.53, 95% CI = 2.56–22.14, *p* < 0.001), Table 3. After adjustment for several well-known cofounders, multivariate Cox analysis showed that the association between high plasma dp-ucMGP levels and the study endpoints remained significant for all-cause mortality (HR = 2.63, 95% CI = 1.17–5.94, *p* = 0.02), CV mortality (HR = 2.82, 95% CI = 1.07–7.49, *p* = 0.037) and progression of CKD (HR = 4.02, 95% CI = 1.20–13.46, *p* = 0.024), whereas the association with CV events was lost. To verify the results in a larger population, we performed the original univariate Cox analysis in 300 bootstrap samples and found that the association between high dp-ucMGP and the composite renal outcome remained significant (HR = 2.02, 95% CI:1.24–3.67, *p* = 0.003, Supplementary Table S1). Plasma ox-LDL failed to show any associations with study end-points or dp-ucMGP.

## 3. Discussion

Diabetic CKD patients are at high risk for developing accelerated atherosclerosis, sudden CV death, and progression of renal disease. This heavy morbidity cannot be solely explained by traditional risk factors. In this study we aimed to investigate the association of plasma dp-ucMGP with OS, early atherosclerosis, CV disease, mortality, and lower renal function in a cohort of patients with diabetic CKD.

Epidemiological data have repeatedly reported a strong, inverse association between vitamin K status and CV mortality and morbidity through inactivation of the VC inhibitor MGP. The Rotterdam study was the first to show that low vitamin K2 intake was associated with severe aortic calcification and increased all-cause and coronary heart disease-mortality in a large cohort of 4,807 community-dwelling subjects with no history of CV disease at baseline [10]. Similarly, data from the population-based Third National Health and Nutrition Examination Survey (NHANES III) and Prevention of Renal and Vascular End-Stage Disease (PREVEND) studies reported a significant inverse association between vitamin K status and CV mortality and morbidity [11,12]. Moreover, it became evident that among all forms of MGP, dp-ucMGP is the fully inactive form, directly related to the development of accelerated VC and indicative of vitamin K status [8,13,14]. Furthermore, decreased vitamin K status was strongly associated with increased circulating dp-ucMGP and subsequent severe arterial calcification in various populations, including diabetics [15], kidney transplant recipients [16], and HD patients [17]. In this study, we found that dp-ucMGP was positively correlated with triglycerides and CRP and negatively with HDL, whereas no association was found with ox-LDL. In agreement with our findings, several studies have reported on the link between circulating dp-ucMGP and inflammation/dyslipidemia in CKD patients [14,16,18].

During the past few decades, OS has emerged as a novel risk factor for CV disease [19,20] and progression of renal function in CKD [21]. Ox-LDL plays a pivotal role in the development of diabetic CKD [22]; not only does the onset of diabetic CKD trigger the generation of ox-LDL molecules [23], but also diabetics with albuminuria exhibit increased plasma ox-LDL levels [24]. Moreover, ox-LDL has been shown to increase in parallel to diabetic CKD progression [25]. In our study, ox-LDL failed to show any association with circulating dp-ucMGP, or with any of our study end-points. Therefore, in this cohort of diabetics with CKD, dp-ucMGP was associated with renal impairment and CV mortality and morbidity through a pathway other than OS (probably through inflammation and VC).

In our study, we found that circulating dp-ucMGP was strongly associated with all-cause and CV mortality in the high atherogenic population of diabetic CKD patients. This finding has been repeatedly highlighted in the general population [26,27] and patients with CV disease or heart failure [28,29]. In a cohort of 518 T2DM patients followed for a median of 11.2 years, among all MGP types, only circulating dp-ucMGP was independently associated with CV disease, heart failure and peripheral arterial disease [15]. In a cross-sectional study that included 198 T2DM patients with normal or slightly impaired renal function, plasma dp-ucMGP was strongly correlated with peripheral arterial calcification score, even after adjustment for several well-established cofounders, such as gender, age and background history of CV disease [30]. Similar results were published in CKD and HD patients. Schurgers et al. measured plasma dp-ucMGP levels at baseline in 107 patients at CKD stages 2–5, followed for 802 ± 311 days with mortality as the main outcome [7]. All patients were divided into two groups according to median dp-ucMGP. Compared to the lower half, patients in the high dp-ucMGP category had an increased risk for all-cause mortality and severe aortic calcification. Likewise, in a cohort of 518 kidney transplant recipients followed for a median of six years, plasma dp-ucMGP was independently associated with all-cause mortality, even after adjustment for potential cofounders such as renal function and treatment with a vitamin K antagonist [31]. Moreover, two separate meta-analyses were conducted to investigate the possible association between dp-ucMGP and CV mortality and morbidity. Chen et al. included 21 studies and 222,592 participants and reported that high plasma dp-ucMGP was strongly associated with increased risk for all cause and CV mortality [32]. Similarly, another meta-analysis of 11 studies and 33,289 patients showed that circulating dp-ucMGP was associated with high risk for total mortality (HR = 1.77) and CV mortality (HR = 1.84), whereas no association was found between dp-ucMGP and CV disease [33]. These findings are in agreement with ours and indicate that circulating dp-ucMGP might be associated with mortality but not CV disease.

Similar to vitamin K, vitamin D is a micronutrient with pleiotropic functions and a metabolic role in calcium homeostasis and VC. Vitamin D deficiency is highly prevalent in CKD and has been repeatedly associated with endothelial dysfunction and VC, probably by downregulating the expression of MGP. In a cross-sectional observational study, Mayer et al. divided 1023 healthy subjects into groups according to vitamin K and D status [assessed by circulating dp-ucMGP and 25-hydroxyvitamin D3 (25-OH-D3), respectively]. The authors found that compared to all other groups, patients with a deficiency of both vitamins had the highest values of arterial pulse wave velocity. Moreover, pulse wave velocity was independently associated with both dp-ucMGP and 25-OH-D3. The authors concluded that a deficiency of both vitamin K and D might act synergistically to promote arterial stiffness [34]. In CKD patients, subclinical deficiency of vitamin D and K status (assessed by circulating dp-ucMGP and 25-OH-D, respectively) was highly prevalent and associated with albuminuria and CKD stage [35]. In a cohort of 461 kidney transplant recipients with mild CKD followed for a period of 9.8 years, decreased vitamin K and D status were strongly associated with increased risk for mortality and graft failure. In a further analysis, the authors stratified all patients according to vitamin D treatment and found that the associations between dp-ucMGP and study endpoints were more pronounced in patients treated with vitamin D, compared to those that did not receive vitamin D [36]. In this study, the authors did not assess whether dp-ucMGP differed significantly among the two groups (vitamin D treated and untreated patients). This was evaluated by Fusaro et al. in a different population of HD patients. The authors found that supplementation with vitamin D was associated with increased levels of circulating vitamin K dependent proteins in HD [37]. Moreover, in a CKD setting, interventions with vitamin D analogues have been shown to improve endothelial dysfunction [38]. Therefore, the interplay between vitamin K and D is more pronounced in uremia and supplementation of active vitamin D compounds might contribute to the preservation of MGP activity and improve vascular health in CKD and HD.

The main finding of our study was that dp-ucMGP was associated with lower renal function in a cohort of diabetic CKD patients. Despite the development of several novel therapies and interventions, compared to other medical specialties, nephrology lags behind in the number and quality of randomized controlled trials [39]. One of the main reasons for that is that in CKD settings, frequently used endpoints include the initiation of renal replacement therapy and/or doubling of serum creatinine (which corresponds to a 57–60% decline in eGFR), which are late events and may not be reached during the study period. Having these in mind, the endpoint that we adopted (a 30% reduction of eGFR) has been recently established as a validated early surrogate endpoint for the progression of CKD [40,41]. In our study, at baseline, plasma dp-ucMGP was significantly correlated with eGFR and proteinuria, a well-known risk factor for CKD progression and mortality [42]. In cohorts similar to ours (CKD at stages 2–5), five studies demonstrated that eGFR was negatively associated with circulating dp-ucMGP [7,9,43,44,45]. Similar results were reported in kidney transplant recipients [16], diabetics with normal kidney function [46] and CKD [30]. Likewise, in a multi-ethnic population study including 1166 white Flemish and 714 South Africans from the general population, eGFR was reduced and the risk for renal function impairment was increased with higher circulating dp-ucMGP levels [47]. In agreement with our results, Wei al. randomly recruited 1009 subjects from the general population and found that plasma dp-ucMGP was independently associated with renal dysfunction (as assessed by a decrease of eGFR and the presence of albuminuria) over a period of 8.9 years. The HRs for eGFR reduction below 60 mL/min and for developing albuminuria were 3.49 and 4.70, respectively [48]. The same group of researchers obtained kidney tissue from biopsies of two healthy kidney donors and four CKD patients and stained all renal samples for calcium deposits and total ucMGP. In normal renal tissues, no calcification was found and ucMGP was not detected; however, in the CKD samples, both microcalcifications and ucMGP were found [49]. Based on these histopathological findings, the authors suggested that ucMGP might promote calcification not only in large arteries—as was repeatedly demonstrated—but in intrarenal vessels as well, highlighting a new molecular pathway for the progression of CKD. In a cohort of 1006 healthy subjects, Jaques et al. reported that dp-ucMGP levels were independently associated with renal resistive index (a marker obtained by renal artery Doppler ultrasound that is associated with renal damage) [50]. Posch et al. conducted a retrospective study of 14,432 patients with atrial fibrillation and CKD stages 3–4 that were followed for five years and reported that in this cohort, treatment with vitamin K antagonists was associated with a faster progression of CKD [51]. These data suggest that downregulation of vitamin K (and subsequently MGP) might favor the deterioration of renal function through the promotion of renal microcalcification. Moreover, in vivo, supplementation with vitamin K1 was found to inhibit the development and progression of nephrolithiasis through the upregulation of MGP in kidney tissues [52]. In rats, after 5/6 nephrectomy, renal expression of MGP was upregulated, and in human kidney tissues obtained from biopsies due to nephrotic syndrome, eGFR was inversely associated with glomerular and tubulointerstitial MGP expression. Similar to our findings, increased MGP expression in the renal tubules and the interstitium was associated with a 3.3-fold higher risk for the composite endpoint of a 40% decline in eGFR and progression to ESRD [41].

CKD is a state of enhanced VC and vitamin K deficiency. Circulating K1 and K2 levels do not necessarily reflect the vitamins’ bioavailability for MGP activation. This is why currently, the most reliable markers of vitamin K status are considered circulating dp-ucMGP and proteins induced by vitamin K absence or antagonism (PIVKA), which is a collective marker of all undercarboxylated, inactive vitamin K dependent proteins [53]. The prevalence of vitamin K deficiency (measured by PIVKA) augments in parallel with progression of CKD [54] and reaches 83–97% in HD patients [35,55]. Moreover, in these populations, subclinical vitamin K deficiency (assessed either by PIVKA or dp-ucMGP) has been repeatedly associated with increased VC and CV disease [56,57].

Although several other investigators have demonstrated an association between circulating dp-ucMGP and well-established renal damage biomarkers such as eGFR, serum creatinine or proteinuria, until recently, the pathophysiological mechanism underlying this association was hypothesized to be that renal damage is a strong determinant of vascular vitamin K depletion. However, in this study, we demonstrated that dp-ucMGP is associated with the progression of diabetic CKD and therefore we propose that vitamin K deficiency might be an independent risk factor for the development and progression of kidney disease through calcium deposition into renal arteries, the formation of kidney stones, the impairment of renal microcirculation, and generalized atherosclerosis.

To the best of our knowledge, this is the first report on the association between dp-ucMGP, OS, and CV mortality/morbidity in a cohort of diabetic CKD patients followed for a long period of seven years. Furthermore, in this population, it is the first study to assess the association between dp-ucMGP and lower renal function after a long period of follow-up. Moreover, the association between dp-ucMGP and ox-LDL, a biomarker of OS and atherogenesis, has not been examined in other studies. However, there are certain limitations that should be addressed. The observational design of our study precludes establishing causality and our study cohort was relatively small, even though in most studies that assessed plasma dp-ucMGP in patients with renal impairment, the sample size was similar to ours, or even smaller [7,9,44,45]. Moreover, the main finding of our study was verified in a larger sample size (bootstrapping for 300). However, our results should be interpreted with caution and further large studies that can establish causality are needed for dp-ucMGP to qualify as a novel risk factor for CV disease and the progression of CKD. Several ongoing randomized controlled trials in CKD and HD patients are currently investigating the effect of vitamin K supplementation on dp-ucMGP levels and hard end-points, including mortality and CV events [58]. However, the preservation of renal function as an endpoint has not yet been assessed.

## 4. Materials and Methods

### 4.1. Patients

A total of 66 Caucasian adult patients with T2DM and CKD were recruited from the Diabetic Nephropathy Department’s outpatient clinic of the University General of Alexandroupolis in Greece. All participants gave their written, informed consent. The protocol of our study was in accordance with the Helsinki Declaration of Human Rights and was approved by the Ethics Committee of the Medical School of the Democritus University of Thrace in Alexandroupolis, Greece (1130/25 November 2011). Inclusion criteria were a history of T2DM of at least 10 years, presence of diabetic retinopathy, persistent albuminuria and eGFR below 90 mL/min. Exclusion criteria included the presence of urinary tract disease, cancer, acute illness, chronic inflammatory disease, treatment with vitamin K antagonists and a documented transitory of permanent decline in eGFR over 30% during the last six months before enrollment. Anthropometric, clinical, and biochemical characteristics and a history of previous CV disease were documented at baseline. The definition and classification of CKD in stages was done according to the criteria established by the Clinical Practice Guidelines for Chronic Kidney Disease from the National Kidney Foundation’s Kidney Disease Outcomes Quality Initiative [59] and eGFR was calculated by the CKD epidemiology collaboration (CKD-EPI) equation [60].

### 4.2. Follow-Up and Endpoints

After the baseline assessment, all patients were prospectively followed for a period of 7 years, or the occurrence of death, or a composite renal outcome of 30% decline in eGFR, or progression to ESRD requiring renal replacement therapy. The secondary outcome was the occurrence of CV events. At the end of the study, renal function was re-assessed with a new estimation of eGFR. Follow-up data were obtained from death certificates, regular follow-up visits, and through integrated telephone interviews.

### 4.3. Laboratory Analyses

Laboratory methods for our study have been described before [25]. Fasting blood samples were collected from all patients to obtain plasma, serum and whole blood. The presence of proteinuria was evaluated as the urine protein (turbidimetric immunoassay) to creatinine ratio in a morning spot urine sample, as described elsewhere [61]. Plasma samples for dp-ucMGP and ox-LDL were centrifuged immediately and stored at −20 °C until analysis. Plasma dp-ucMGP levels were determined as described by Schurgers et al. [7] by using a sandwich enzyme-linked immunosorbent assay (ELISA) which involved 2 anti-MGP monoclonal antibodies. The detection antibody is directed against the un-carboxylated MGP sequence 35 to 49 (mAb-ucMGP; VitaK BV, Maastricht, The Netherlands), whereas the antibody directed against the non-phosphorylated MGP sequence 3 to 15 is used as the capture antibody (mAb-dp-MGP; VitaK BV). As a standard, we used the synthetic peptide dp-MGP3-15- (AADO)-ucMGP35-54. The intra- and inter-assay variability values were 5.6% and 9.9%, respectively. Plasma levels of ox-LDL were determined by ELISA (human ox-LDL ELISA kit, Mercodia, Sweden). Detection limits for ox-LDL assays were 0.3 U/L, according to the manufacturers. Intra- and inter-assay coefficients of variation were < 10%.

### 4.4. Statistics

Statistical analyses were carried out using the IBM Statistical Package for Social Sciences (SPSS 18.0 for Windows, Chicago, IL, USA). To assess the association between plasma dp-ucMGP and study outcomes, all patients were divided into 2 groups according to the median dp-ucMGP value (i.e., below or equal and above 656 pM). The Kolmogorov–Smirnov test was used to test data for normality. Variables were expressed as the mean (standard deviation) or median (range), as appropriate. Differences among groups were established using the Mann-Whitney test for continuous variables and the chi-square test for dichotomized variables. Bivariate associations between variables were examined with Spearman’s correlation co-efficient. Estimating a 30% occurrence of the composite renal endpoint over 7 years in our population with diabetic pre-dialysis CKD, as described in a recent study in a population similar to ours [62], we calculated a sample size of at least 62 patients to give approximately 80% power (alpha = 0.05, two-tailed) to reject the null hypothesis. To estimate overall survival, CV survival, CV events, and the renal composite outcome for the median plasma dp-ucMGP level, we used the Kaplan–Meier actuarial method and the log-rank test to compare survival curves. Univariate and multivariate survival analyses were performed using Cox proportional hazard analysis to evaluate adjusted HRs and 95% CIs for the associations between groups of dp-ucMGP and study outcomes. The models for the combined renal outcome of a 30% decline in eGFR and/or development of ESRD were adjusted for all variables which were associated with this outcome in the univariate analysis (serum albumin, proteinuria, and duration of T2DM). The variables that were incorporated into the CKD-EPI formula for eGFR estimation were not included in this analysis. The univariate Cox analysis regarding renal outcome was also performed in 300 bootstrap samples. The models for all-cause/CV mortality and morbidity were adjusted for well-established confounding factors, that were associated with these outcomes in the univariate analysis, including age, sex and BMI. Significance was set at *p* < 0.05.

## 5. Conclusions

Circulating vitamin K dependent dp-ucMGP is strongly associated with all-cause/CV mortality and lower renal function in diabetic CKD. The pathophysiologic mechanism underlying this association is not by implication of OS, but probably by the promotion of renal microcalcification and/or inflammation. Dp-ucMGP might be a novel, promising therapeutic target for inhibiting progression of CKD by reversing vitamin K deficiency. Further large, prospective, randomized controlled trials are needed in order to draw definite conclusions.

## Figures and Tables

**Table 1:** Anthropometric, clinical and biochemical characteristics of diabetic kidney disease patients below and above median plasma dpucMGP levels. Results for continuous variables are presented as mean (standard deviation (SD)) or median (range).

	Dp-ucMGP < 656 pM	Dp-ucMGP ≥ 656 pM
*n* = 66	33	33
dp-ucMGP (pM)	351 (87–655)	1125 (657–2743)
Age (years)	67.4 ± 8.6	69.7 ± 8.1
Gender (M/F)	17/16	18/15
BMI (kg/m^2^)	31.3 ± 4.6	31.9 ± 5.3
Duration of T2DM (years)	16.0 ± 9.2	16.2 ± 7.5
Duration of HP (years)	14.9 ± 7.9	15.8 ± 7.3
History of CV disease (yes)	10/33	6/33
Hemoglobin (g/dL)	12.6 ± 1.6	11.8 ± 2.1
Albumin (g/dL)	4.3 ± 0.4	4.0 ± 0.5
eGFR at baseline (mL/min)	64.4 ± 24.6	35.1 ± 12.7
Proteinuria	0.16 (0.02–2.1)	0.40 (0.01–7.0)
Triglycerides (mg/dL)	120 (53–450)	189 (89–310)
Total cholesterol (mg/dL)	178.7 ± 37.3	190.6 ± 44.4
LDL-cholesterol (mg/dL)	98.8 ± 28.5	112.2 ± 37.4
HDL-cholesterol (mg/dL)	50.3 ± 15.9	42.5 ± 8.9
HbA1c (%)	7.5 ± 1.3	7.5 ± 1.1
CRP (mg/dL)	0.5 ± 0.9	0.9 ± 1.0
Ox-LDL (U/L)	59.5 ± 17.7	65.4 ± 16.5

Mann–Whitney test or chi-square for differences of variables among groups. Dp-ucMGP, dephosphorylated uncarboxylated matrix Gla protein; BMI, body mass index; T2DM, type 2 diabetes mellitus; HP, hypertension; CV, cardiovascular; eGFR, estimated glomerular filtration rate; LDL, low-density lipoprotein; HDL, high-density lipoprotein; HbA1c, glycated hemoglobin A1c; CRP, C-reactive protein; ox-LDL, oxidized low-density lipoprotein.

**Table 2:** Correlation matrix between dp-ucMGP and age, sex, BMI, duration of T2DM, hypertension, hemoglobin, triglycerides, total cholesterol, LDL-cholesterol, HDL-cholesterol, HbA1c, CRP, albuminuria, eGFR at baseline and at the end of the follow-up period, and ΔeGFR. Values represent Spearman’s correlation coefficients.

	Dp-ucMGP	
	*r*	*p*
Age	0.17	0.18
Sex	−0.15	0.25
BMI	0.13	0.31
Duration of T2DM	0.11	0.38
Duration of Hypertension	0.91	0.47
Hemoglobin	−0.13	0.31
eGFR at baseline	−0.69 ^b^	**<0.0001**
eGFR after follow-up	−0.72 ^b^	**<0.0001**
ΔeGFR	−0.51 ^b^	**<0.0001**
Albumin	−0.30 ^a^	**0.02**
Proteinuria	0.40 ^b^	**0.002**
Triglycerides	0.28 ^a^	**0.03**
Total cholesterol	−0.05	0.67
LDL-cholesterol	0.01	0.96
HDL-cholesterol	−0.22	0.08
HbA1c	0.21	0.09
CRPO x-LDL	0.28 ^a^ 0.08	**0.03** 0.54

^a^ Correlation is significant at the 0.05 level. ^b^ Correlation is significant at the 0.01 level.

**Table 3:** Cox proportional hazard analysis (forward stepwise regression) showing the association of dp-ucMGP with all-cause mortality, cardiovascular mortality, fatal/non-fatal cardiovascular events, and deterioration of renal function in univariate and multivariate models.

All-Cause Mortality			
		HR	CI
Model 1 ^a^			
	dp-ucMGP ≥ 656 pM	2.68	1.21–5.94
Model 2 ^b^			
	dp-ucMGP ≥ 656 pM	2.63	1.17–5.94
**Cardiovascular Mortality**			
Model 1 ^a^			
	dp-ucMGP ≥ 656 pM	2.86	1.10–7.47
Model 2 ^b^			
	dp-ucMGP ≥ 656 pM	2.82	1.07–7.49
**Cardiovascular Events**			
Model 1 ^a^			
	dp-ucMGP ≥ 656 pM	2.03	1.02–4.02
Model 2 ^b^			
	dp-ucMGP ≥ 656 pM	1.84	0.91–3.73
**≥30% eGFR Reduction or Progression to ESRD**			
Model 1 ^a^			
	dp-ucMGP ≥ 656 pM	7.53	2.56–22.14
Model 2 ^b^			
	Proteinuria	1.54	1.04–2.27
	dp-ucMGP ≥ 656 pM	4.02	1.20–13.46

Model 1 ^a^ = univariate model. Model 2 ^b^ for all cause/cardiovascular mortality and cardiovascular events = multivariate model, adjusted for age, sex, body mass index; Model 2 ^b^ for ≥30% eGFR (estimated glomerular filtration rate) reduction or progression to end-stage renal disease (ESRD) = multivariate model, adjusted for duration of T2DM, serum albumin and proteinuria. HR = hazard ratio, CI = confidence interval.

## Supplementary Materials

Supplementary materials can be found at https://www.mdpi.com/1422-0067/21/17/6035/s1. Table S1: Univariate Cox proportional hazard analysis in 300 bootstrap samples, showing the association between high dp-ucMGP and the composite renal outcome of at least 30% eGFR reduction or progression to ESRD.

## Abbreviations

25-OH-D3	25-hydroxyvitamin D3
ΔeGFR	Change in estimated glomerular filtration rate
BMI	Body mass index
CKD	Chronic kidney disease
CKD-EPI	Chronic kidney disease epidemiology collaboration equation
CI	Confidence interval
CRP	C-reactive protein
CV	Cardiovascular
Dp-ucMGP	Dephosphorylated, uncarboxylated matrix Gla protein
eGFR	Estimated glomerular filtration rate
ESRD	End stage renal disease
HBA1c	Glycated hemoglobin
HD	Hemodialysis
HDL	High-density lipoprotein
HR	Hazard ratio
LDL	Low-density lipoprotein
MGP	Matrix Gla protein
NHANES	National Health and Nutrition Examination Survey
OS	Oxidative stress
Ox-LDL	Oxidized low-density lipoprotein
PIVKA	Proteins induced by vitamin K absence or antagonism
PREVEND	Prevention of renal and vascular end stage disease
T2DM	Type 2 diabetes mellitus
VC	Vascular calcification
