# Supplementary material for: The Association of dp-ucMGP with Cardiovascular Morbidity and Decreased Renal Function in Diabetic Chronic Kidney Disease
Source: Int J Mol Sci. 2020 Aug 21;21(17):6035. doi: 10.3390/ijms21176035 (PMC7504709; doi:10.3390/ijms21176035)
Supplement: Supplementary file 1 [file ijms-21-06035-s001.pdf]

Supplementary Table S1. Univariate Cox proportional hazard analysis in 300 bootstrap samples, showing the association between high dp-ucMGP and the composite renal outcome of at least 30% eGFR reduction or progression to ESRD.

≥30% eGFR reduction or progression to ESRD

	Hazard Ratio	95% Confidence Interval	P
Dp-ucMGP ≥656pM	2.02	1.24-3.67	**0.003**
